# MicroRNA-30a-3p acts as a tumor suppressor in MHCC-97H hepatocellular carcinoma cells by targeting COX-2

**DOI:** 10.7150/jca.52298

**Published:** 2021-05-10

**Authors:** XueMei Yang, JiaLing Sun, HaiTao Sun, Bin Wen, MingJia Zhang, HaiYan An, WeiCong Chen, WenTing Zhao, XiaoDan Zhong, ChunYu He, Jie Pang, SongQi He

**Affiliations:** 1 School of Traditional Chinese Medicine, Southern Medical University, Guangzhou, Guangdong, China.; 2 Department of Traditional Chinese Medicine, The Air Force Hospital Of Southern Theater Command, Guangzhou, Guangdong, China.

**Keywords:** Hepatocellular Carcinoma (HCC), MHCC-97H, miR-30a-3p, COX-2.

## Abstract

MicroRNAs (miRNAs) are small, noncoding RNAs which can bind to target mRNAs and regulate gene expression. Increasing evidences suggest that miRNAs play an important role in driving hepatocellular carcinoma (HCC) progression by regulating tumor cell proliferation, apoptosis, invasion, and migration. In this study, we demonstrated that the expression of microRNA-30a-3p (miR-30a-3p) was reduced in HCC cell lines in comparison to immortalized liver cell line, LO2. Augmented miR-30a-3p level markedly inhibited MHCC-97H cell growth, migration and invasion* in vitro*. MiR-30a-3p was also found to inhibit tumor growth *in vivo* using tumor-bearing mice. Mechanismly,* COX-2* was discovered to be a direct and functional target of miR-30a-3p in MHCC-97H cells. Raised miR-30a-3p expression reduced the transcriptional level of *COX-2* in MHCC-97H cells, while genetically upregulated *COX-2* expression was able to reverse the function of miR-30a-3p-mediated suppression of MHCC-97H cells growth, migration and invasion. In addition, we found that using a COX-2 inhibitor, celecoxib, could enhance the anti-metastatic role of miR-30a-3p in MHCC-97H cells. Lastly, we found that decreased COX-2 protein level affected PGE2 production, leading to lower Bcl-2, Caspase-3, MMP2 and MMP9 expression but higher Bax and E-cadherin expression, which in turn culminated in higher rates of cell death and lower rates of cell migration. Taken together, our findings demonstrate that miR-30a-3p could be a target for the treatment of hepatocellular carcinoma cells progression.

## Introduction

Hepatocellular carcinoma (HCC) is the most common diagnosed primary liver malignancy 1. While the overall survival of HCC patients has improved over the past two decades with the development of increasingly advanced diagnostic and therapeutic modalities, these patients still suffer from significantly poor prognosis which may be attributed to increased rates of tumor recurrence and metastasis 2. Therefore, it is essential to gain a deeper understanding of the molecular mechanism underlying HCC to promote the development of novel therapeutic strategies.

MicroRNAs (miRNAs) are small, single-stranded, noncoding RNAs of approximately 19 to 22 nucleotides in length. These molecules bind to their target mRNAs, resulting in mRNAs silencing or post-transcriptional regulation 3, 4. Circulating miRNAs may function as diagnostic and prognostic biomarkers for HCC 5. Previous studies have indicated that miR-612, miR-34 and miR-191 play a potentially significant role in HCC 6-8. MiR-612 has been found to negatively regulate HCC cell invasion, migration and proliferation, and its expression has been found to be inversely correlated with tumor metastasis, epithelial-mesenchymal transition (EMT), tumor stage and size 8. Moreover, specific targeting of miR-34a using a liposome-formulated mimic has entered a multicenter clinical trial to evaluate safety and effectivity in patients with primary liver cancer or with liver metastasis from other cancers 6. Evidently, miRNAs may not only regulate the biological behavior of HCC, but may also function as disease biomarkers and therapeutic targets.

MiR-30a-3p belongs to the miR-30 family and has been reported to be downregulated across several types of malignancies 9. Previous reports revealed that miR-30a-3p suppressed LADC EKVX lung adenocarcinoma cell line migration and proliferation 10 and was also able to slow the proliferation of HepG2 and Bel-7402 HCC cell lines 11, 12. Nevertheless, the role of miR-30a-3p in HCC has yet to be fully documented.

In this study, we tried to uncover the function of miR-30a-3p in MHCC-97H HCC cell line. We observed a remarkable downregulation of miR-30a-3p level across three different HCC cell lines. MiR-30a-3p overexpression was found to significantly suppress MHCC-97H cell growth both *in vivo* and *in vitro*. Additionally, overexpression of miR-30a-3p suppressed MHCC-97H invasion and migration *in vitro*. We identified *COX-2* as a potential direct target of miR-30a-3p in MHCC-97H cells. Elevated miR-30a-3p expression appeared to reduce *COX-2* expression levels. Furthermore, we also discovered that COX-2 overexpression was able to partially reverse the effects of miR-30a-3p in MHCC-97H cells.

## Materials and methods

### Cell culture and animals

HCC cell lines (MHCC-97L, MHCC-97H, Hep3B) and immortalized liver cell line (LO2) were procured from KeyGEN BioTECH (JiangSu, China). All cells were maintained in 10% fetal bovine serum (FBS, Gibco Life Technologies, Grand Island, NY, USA) supplemented with high glucose Dulbecco's modified Eagle's medium (DMEM, Invitrogen, Carlsbad, CA, USA) with the addition of 100 U/mL penicillin/streptomycin (Invitrogen, Carlsbad, CA, USA). All cultures were maintained in humidified environments of 5% CO_2_ at 37°C.

All animal experimental procedures were reviewed and approved by the Southern Medical University Animal Use and Care Committee (Guangdong, China). Female immunodeficient BALB/c-nu/nu mice weighing 20±2 g between 6 to 8 weeks of age were obtained from the Southern Medical University Medical Laboratory Animal Center and reared under specific-pathogen-free (SPF) conditions under a 12 h light/dark cycle. Food and water were provided *ad libitum*.

### Microarray data processing

Immortalized liver cell line LO2 and HCC cell line MHCC-97H were cultured for total RNA extraction, which was performed utilizing the TRIzol reagent (Invitrogen, Carlsbad, CA, USA). miRNA differential expressions were analyzed with the Agilent Human miRNA (8*60K) array ((Santa Clara, CA, USA) in accordance to preexisting protocols. Fold-change filtering was used to determine differentially expressed miRNAs (*P* < 0.05 and log_2_ [fold change] >1 or <-1).

### Lentivirus transfection

Lentiviruses containing GFP-miR-30a-3p, GFP-miRNA control, GFP-COX-2 mRNA, and GFP-mRNA control were purchased from Genechem Corporation (Shanghai, China) to construct cell lines which stably expressed the aforementioned miRNAs and mRNA. Lentiviral-based transfection was carried out based on pre-determined MOI. Stable cell lines were selected via exposure to 8 μg/mL puromycin (Sigma, USA) and allowed to further proliferate. Quantitative real-time PCR (qRT-PCR) was used to verify that the cultured cell lines overexpressed miR-30a-3p or COX-2.

### qRT-PCR

TRIzol reagent (Invitrogen, Carlsbad, CA, USA) was used for total RNA extraction. cDNA was reversed transcribed using 1 μg of RNA and the PrimeScript^TM^ RT reagent Kit incorporating the gDNA Eraser (RR047A; TaKaRa, Japan). MiRNA and mRNA expressions were detected with the TB Green^®^ Premix Ex Taq™ II (Takara, Japan). Primer sequences are depicted in Table S1. mRNA expression was normalized against GAPDH while miRNA was normalized against U6 expressions. The 2^-ΔΔCt^ method was used to determine relative gene and miRNA expressions.

### Analysis of cell viability and colony formation

Stably transfected MHCC-97H cells which overexpressed miR-30a-3p or control miRNA were seeded onto 96-well plates at a density of 5000 cells per well. Cell viability was determined using the Cell Counting Kit-8 (CCK8, Dojindo Laboratories, Kumamoto, Japan) assay. For colony formation assays, cells were seeded onto 24-well plates (200 cells/well) post-transfection and were subjected to 14 days of culture in complete medium containing 10% FBS at 37°C. After this, cells were fixed using methanol/acetic acid before being stained with 0.5% crystal violet. An inverted microscope was used to quantify the number of colonies which contained >50 cells.

### Xenograft tumor growth

A tumor mouse model was created by subcutaneously injecting MHCC-97H cells stably transfected with miR-30a-3p or miRNA control cells into the right flank of 6-week-old female nude mice (5×10^6^ cells/mouse, 8 mice/group). Vernier calipers were used to measure the tumor sizes every 3 days. Mice were also weighed every 3 days. All experimental mice were euthanized on day 27. Tumors were then dissected and weighed. Tumor volume (cm^3^) was measured as a product of d^2^ × D/2 (d and D represent the shortest and longest tumor diameters, respectively).

### Analysis of apoptosis

Cell apoptosis was detected using the Hoechst 33342/PI staining method (CA1120; Solarbio, China). Briefly, all cells were seeded onto 96-well plates after transfection. These cells were then rinsed twice with cold PBS and subjected to 15 min of staining in the dark with Hoechst 33342 at room temperature. These cells were then exposed for 2 min to propidium iodide (PI) for counterstaining. The cell apoptotic index was determined by observation of five randomly selected fields.

### Cell cycle assays

A 6-well plate was used to house cells at a density of 1×10^6^ cells/well. Cells were detached from the wells at 48 h by trypsin incubation. They were then rinsed with PBS and exposed overnight to 70% (v/v) ethanol at 4°C prior to incubation with RNase and PI for 30-60 min in the dark at room temperature (KGA512, KeyGEN China). Flow cytometry (BD FACSCalibur, BD Biosciences, San Diego, CA, USA) was used to analyze these cells, with the cell cycle phase generated using the FlowJo v10.3 software.

### Wound healing and Transwell assay

A wound healing assay was performed to assess cell migration. Cells were placed into 6-well plates at 5×10^5^ cells per well and grown until a confluent cell monolayer was achieved. The surface of this layer was then gently scratched using a 20-μL pipette tip. Detached cells were washed off using PBS, followed by the addition of growth medium (1%FBS) to allow cell regrowth. Scratched areas were imaged at 0, 24, 48, 72, and 96 h to determine the rate of wound-healing in terms of percentage (%).

To investigate the ability of cells to invade, a Transwell chamber that contained an upper chamber coated with Matrigel (BD Biosciences DiscoveryLabware, Woburn, MA, USA) was used. Serum-free medium was first used to rehydrate the chambers for 2 h at 37°C. 200 μL of cell suspension (containing 6×10^5^ cells) was then inserted in the uppermost chamber. Chemoattractant in the form of 20% FBS supplemented media was added into the bottom chambers. The chambers were then incubated at 37°C for 72 h. After this period, formaldehyde was used to fix the cells present on the lower surface of the membrane at room temperature for 5 min before crystal violet was used to stain the cells for 20 min. The prepared cells were then rinsed thrice with clean water before they were counted under a microscope.

### Enzyme-linked immunosorbent (ELISA) assay

Cells were placed in 6-well plates at a density of 80%-90% and subsequently the supernatant was collected after 24 h of incubation in serum-free DMEM. Cellular debris was removed via centrifugation before samples were frozen at -80°C. A PGE2 ELISA Assay kit (JLC5387; R&D Systems, Inc., Minneapolis, MN, USA) was then used to quantify the amount of prostaglandin E2 (PGE2) in the substance, based on protocols stipulated by the manufacturers.

### Western blotting

Standard western blot analysis was performed. The M-PER Mammalian Protein Extraction Reagent (Thermo Scientific, Rockford, IL, USA) was used to extract cell proteins. A 10% sodium dodecyl sulfate-polyacrylamide gels (SDS-PAGE) was used to separate component proteins before they were immunoblotted onto polyvinylidene difluoride membranes (Millipore, Billerica, MA, USA). Primary antibodies (Bax, #bs-0127M; Bcl-2, #bs-0032R; Caspase-3, #9662; E-cadherin, #ab15148; MMP2, #40994; MMP9, #13667; GAPDH, #5174) were then added to the membranes overnight at 4°C or for 1-2 h at room temperature. Incubation with secondary antibodies took place the following day for 1 h at room temperature. An enhanced chemiluminescence kit (Millipore, Billerica, MA, USA) was used to detect proteins. Protein bands were then detected using a Mini Chemi (Sage Creation, Beijing, China). The ImageJ software (Bio-Rad, Laboratories, Inc., Hercules, CA, USA) with β-actin or GAPDH as loading control was then used to quantify protein levels.

### Statistical analysis

The GraphPad Prism 7.0 (GraphPad, Inc., La Jolla, CA, USA) program was used for all statistical analyses. The results are depicted in terms of mean ± standard deviation (SD). The student's t-test or one-way ANOVA test were used to determine statistical significance between groups, according to the homogeneity of variances. Statistical significance was achieved when *P*<0.05.

## Results

### MiRNA expression profile between MHCC-97H and LO2 cells

**Figure 1:**
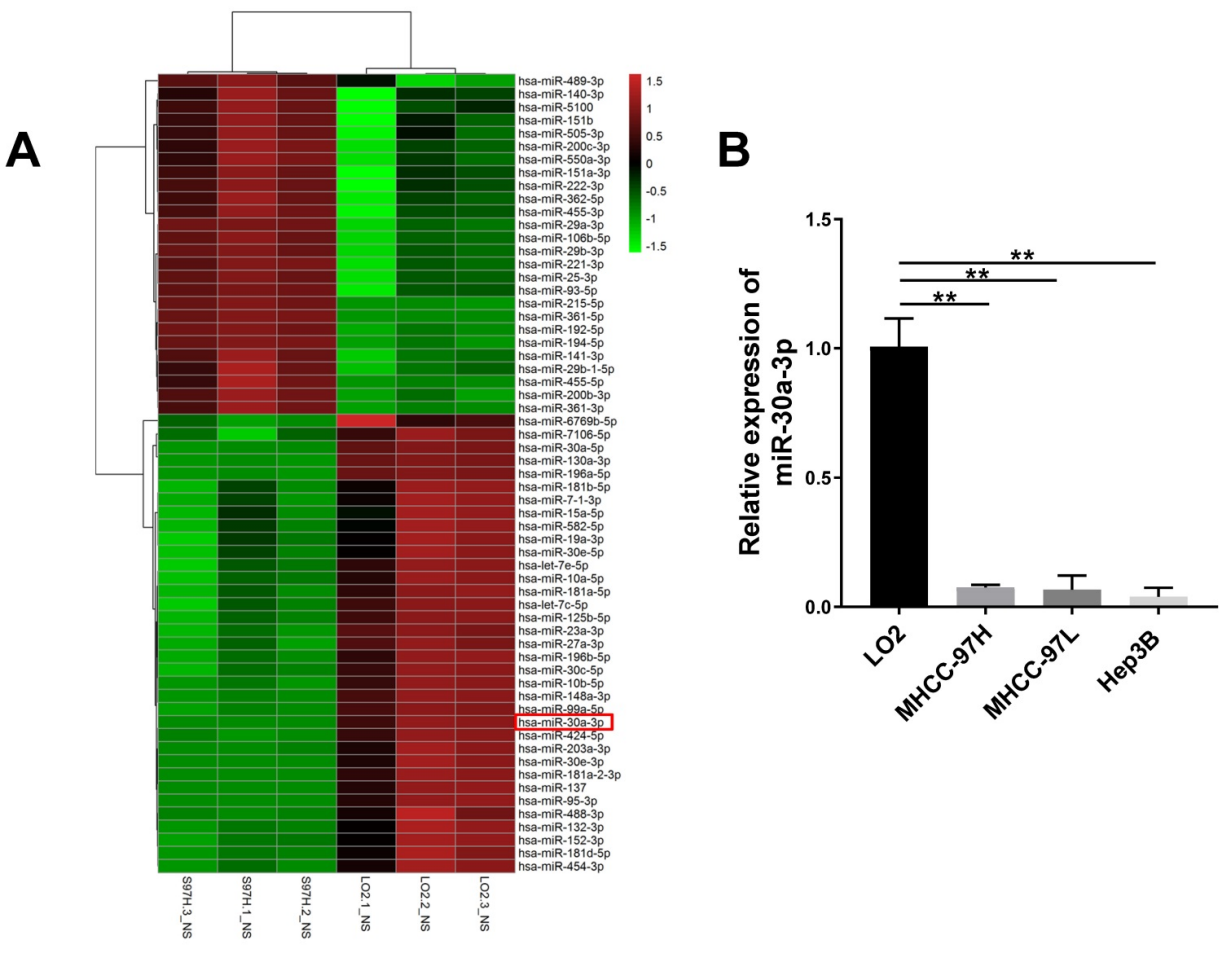
** miRNA expression profile between MHCC-97H and LO2 cells.** (A) Differential microRNAs expression (*P* < 0.05 and log_2_ [fold change] >1 or <-1) were quantified between LO2 and MHCC-97H through microRNA profiling. (B) miR-30a-3p is downregulated in HCC cells (MHCC-97H, MHCC-97L and Hep3B) compared to LO2 cells as assessed using qRT-PCR. Data is presented in terms of mean ± SD, **p<0.001.

First, analysis of miRNA expression profile between MHCC-97H and LO2 cells lines was conducted. A total of 61 distinguishable miRNAs were identified (*P<*0.05, and log_2_ [fold change] >1 or <-1). Among them, 35 miRNAs were downregulated and 26 miRNAs were upregulated in MHCC-97H cells in contrast to LO2 cells (Fig. 1A). Of those 26 miRNAs, 5 of them belonged to the miRNA-30 family, including miRNA-30a-3p, miRNA-30a-5p, miRNA-30e-3p, miRNA-30e-5p and miRNA-30c-5p. Previous studies have established the relationship between miRNA-30a-5p, miRNA-30e-3p, miRNA-30e-5p, miRNA-30c-5p and HCC 13-16. However, the specific functions and mechanisms of miR-30a-3p in HCC still need to be clarified.

The expression of miR-30a-3p across three different HCC cell lines MHCC-97H, MHCC-97L and Hep3B was assessed using qRT-PCR. Reduced miR-30a-3p expressions were noted across the three HCC cell lines in contrast to the immortalized liver cell line LO2 (*P* < 0.005) (Fig. 1B). These findings are consistent with the miRNA sequencing analysis (*P* < 0.005).

### MiR-30a-3p inhibited MHCC-97H cell growth *in vitro and in vivo*

**Figure 2:**
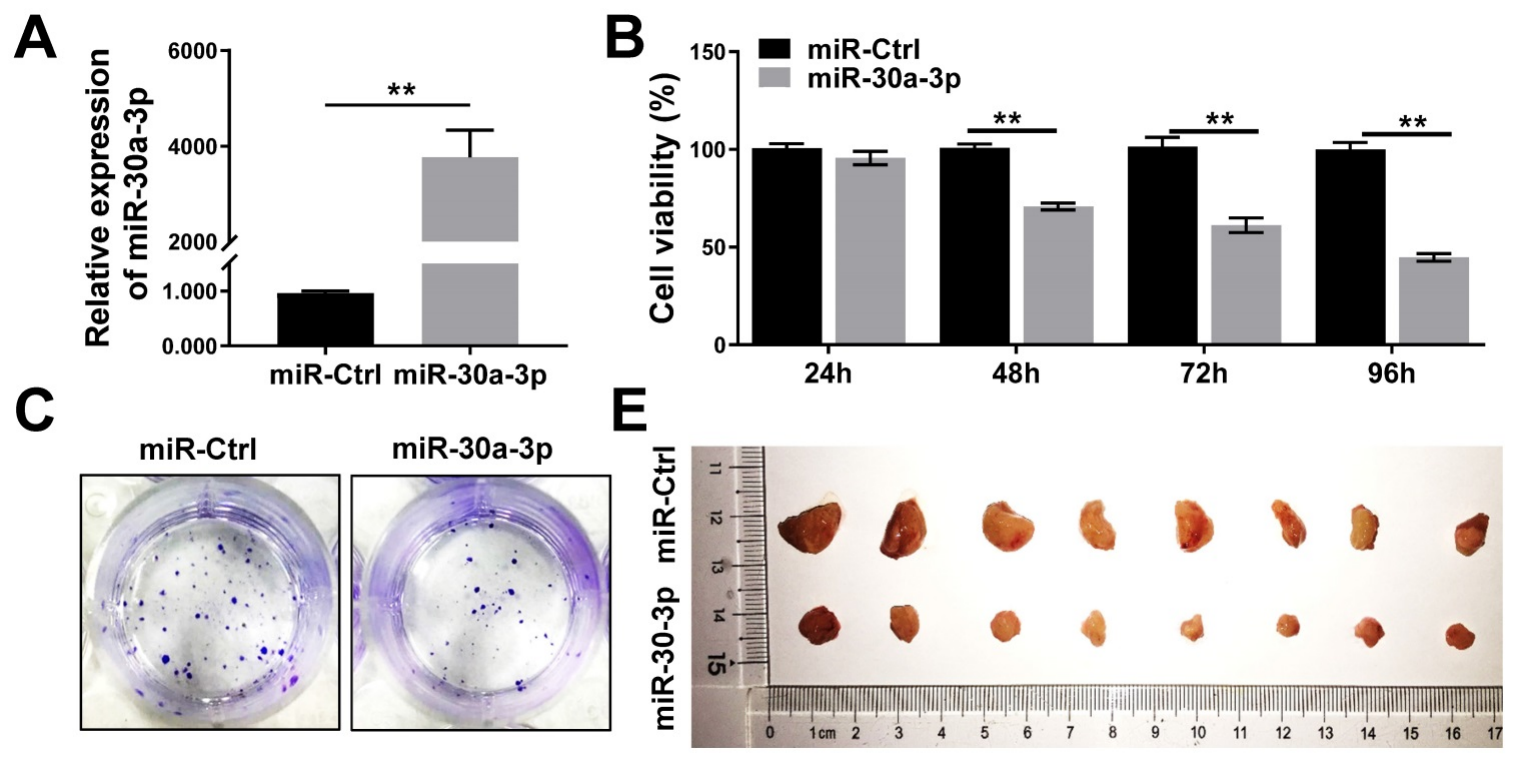

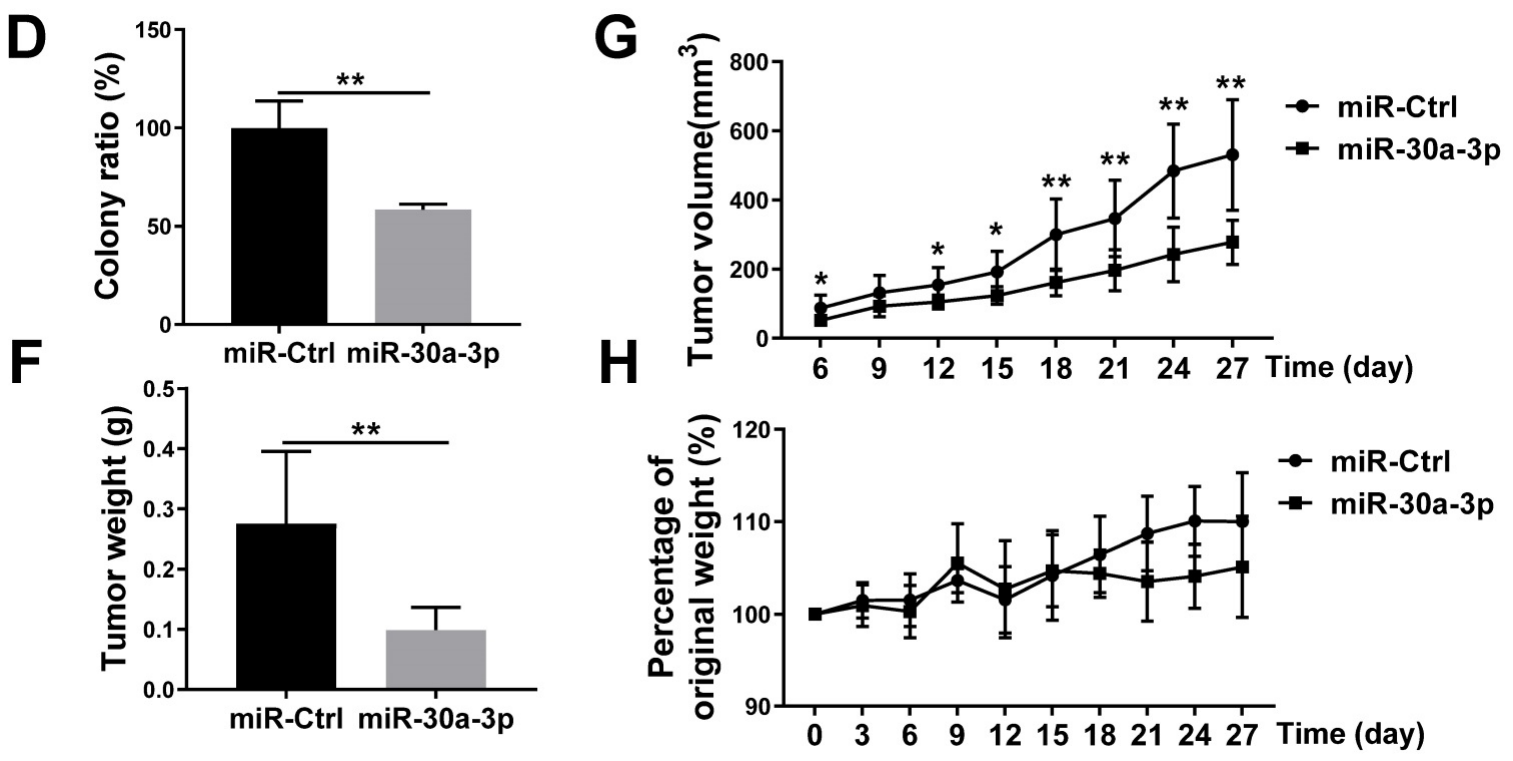
** miR-30a-3p inhibited MHCC-97H cells growth *in vitro* and *in vivo*.** (A) The relative expression of miR-30a-3p was detected by qRT-PCR in MHCC-97H cells transfected with miR-30a-3p or miR-Ctrl. (B) CCK8 assays were performed to measure MHCC-97H cells viability following transfection with miR-30a-3p or miR-Ctrl. (C-D) Cells adhesion was assessed using colony formation assays. 14 days after cell seeding, the colonies were counted after crystal violet staining. Stained colonies with a diameter greater than 1 mm were counted. (E) Representative xenograft tumors derived from MHCC-97H cells between control and miR-30a-3p overexpressed group (n=8 each group). (F) Tumor weights were evaluated when mice were sacrificed at day 27 after subcutaneous inoculation of MHCC-97H cells. (G) Tumor volumes were assessed every 3 days. (H) Body weights measurement was done every 3 days. Data is presented in terms of mean ± SD. **P < 0.01, and *P < 0.05 vs controls.

MHCC-97H cells were transfected with lentiviruses carrying miR-30a-3p or miRNA control (hereafter miR-Ctrl). The efficiency of miR-30a-3p transfection was validated using qRT-PCR (Fig. 2A). CCK8 assays were used to determine the impact of miR-30a-3p on the growth of MHCC-97H cells *in vitro*. Genetically enhanced miR-30a-3p expression significantly suppressed MHCC-97H cells viability in contrast to miR-Ctrl at different time points of 48, 72, and 96 h (Fig. 2B). In line with results achieved using the CCK8 assay, augmented miR-30a-3p expression suppressed the colony formation ability of MHCC-97 cells (Fig. 2C-2D). miR-30a-3p overexpressing MHCC-97H cells were then implanted into immunodeficient BALB/c-nu/nu mice to study *in vivo* tumor growth (8 mice/group). Mice were weighed and tumor volumes were measured every 3 days until day 27. Interestingly, the average tumor volumes were markedly smaller in mice bearing cells from the miR-30a-3p overexpression group compared to the control mice (Fig. 2E-2G). Tumor weights were also heavier in the control group (Fig. 2F). However, mice body weights did not have significant difference (Fig. 2H). Taken together, our findings revealed that miR-30a-3p inhibited HCC cell growth both *in vitro* and *in vivo.*

### MiR-30a-3p induced MHCC-97H cells apoptosis

**Figure 3:**
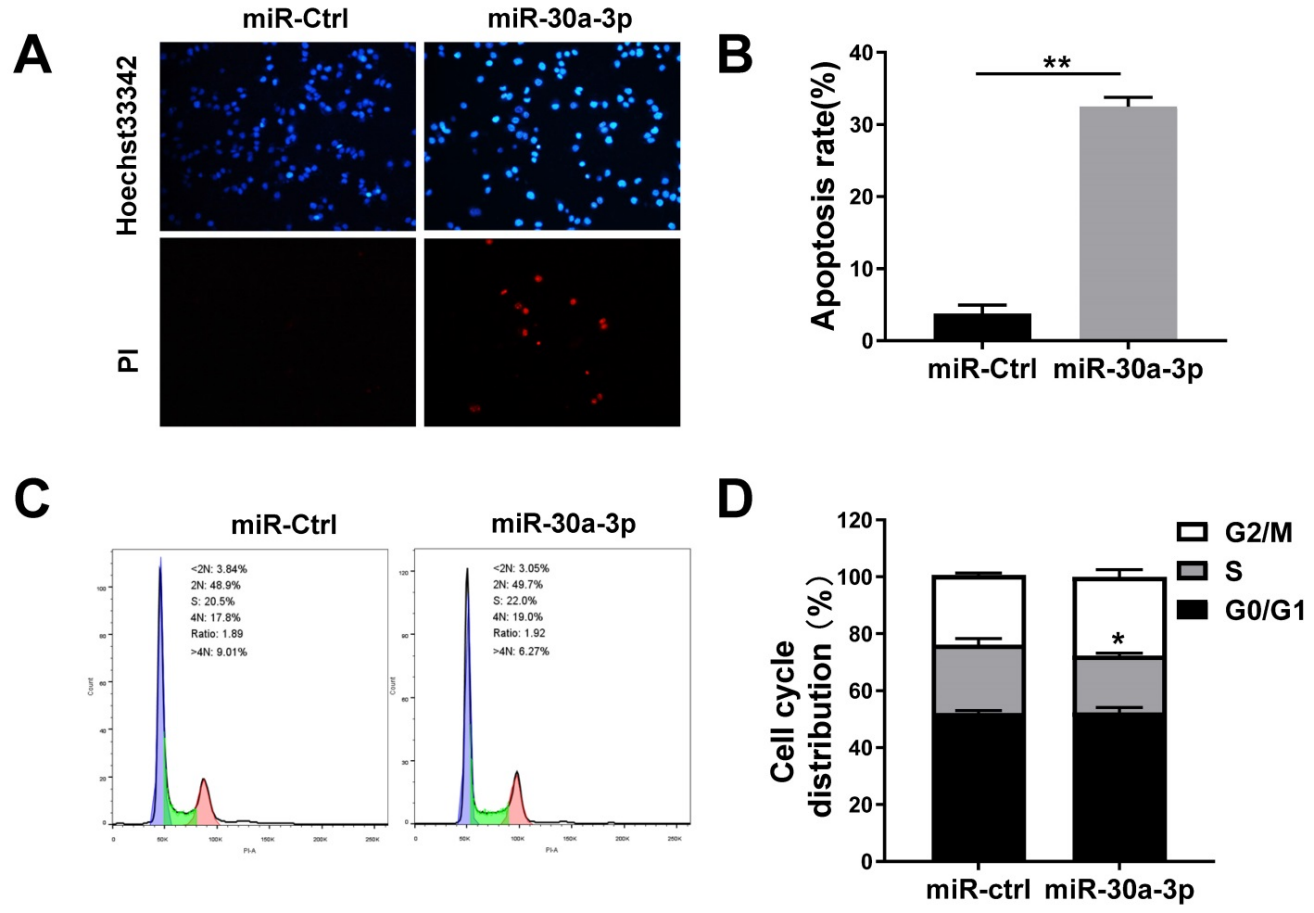
** miR-30a-3p induced MHCC-97H cells apoptosis.** (A-B) Percentage of apoptotic cells determined by Hoechst 33342/ PI staining after incubation for 48 h. (C-D) PI staining allowed for evaluation of cell cycle proportion using Flow cytometry after a 48 h incubation period. Data is presented in terms of mean ± SD of three replicates. *P<0.05, **P<0.01, vs miR-Ctrl.

Previous studies have noted that miR-30a-3p overexpression provoked higher rates of HCC Bel-7402 cells apoptosis 17. In our study, the Hochest33342/PI fluorescent staining assay was used to determine whether miR-30a-3p overexpression induced MHCC-97H cell apoptosis. Our data showed that miR-30a-3p induced apoptosis in MHCC-97H cells after incubation for 48 hours (Fig. 3A-3B).

Given that cell growth represents a balance between cell death and cell proliferation 18, we sought to determine the impact of miR-30a-3p on MHCC-97H cell proliferation. Cells were first cultured for 48 h in complete medium before being subjected to cell cycle analysis using PI staining. While no marked differences existed between the proportion of cells in G0/G1 phase and G2/M phase, there appeared to be smaller proportions of miR-30a-3p overexpressing MHCC-97H cells in S stage (Fig. 3C-3D). We conclude that cell growth is inhibited by miR-30a-3p primarily through its effect on enhancing cell apoptosis.

### MiR-30a-3p inhibited migration and invasion of MHCC-97H cells *in vitro*

**Figure 4:**
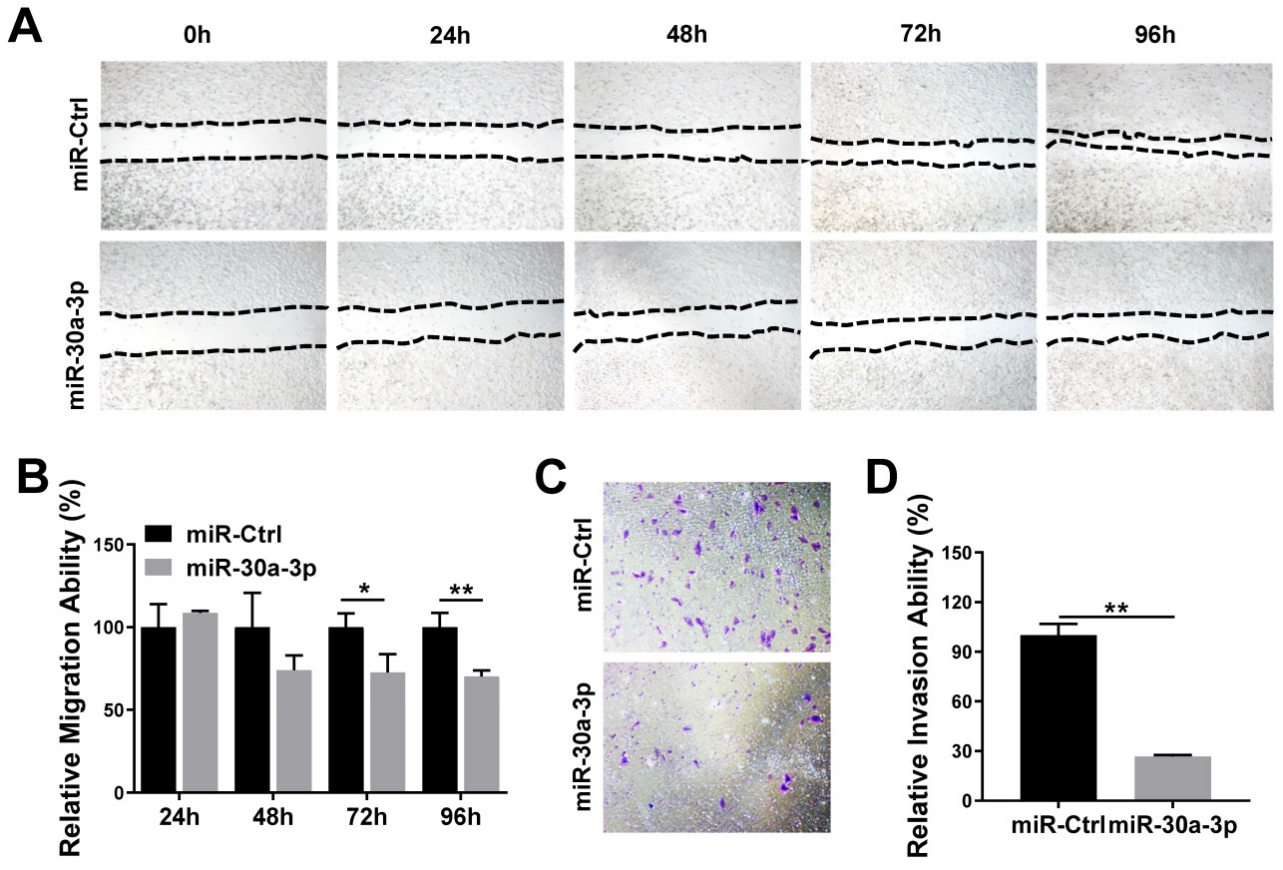
** miR-30a-3p suppressed invasive and migratory abilities of MHCC-97H cells.** (A-B) Wound-healing assay of MHCC-97H cell transfected with miR-30a-3p or miR-Ctrl. Wounds inflicted on a confluent cell monolayer was imaged at 0, 24, 48, 72, and 96 h. (C-D) Transwell invasion assay of MHCC-97H transfected with miR-30a-3p or miR-Ctrl. Cells were cultured on 8 μm Transwell Chambers for 72 h. Data is depicted in terms of mean ± SD (n=3). *P<0.05, **P<0.01 vs miR-Ctrl.

We studied the impact of miR-30a-3p on HCC cell migration and invasion using the wound-healing and Transwell Matrigel invasion assays, respectively. Wound healing assays demonstrated that MHCC-97H cells which overexpressed miR-30a-3p had markedly decreased migratory abilities in contrast to miR-Ctrl cells (Fig. 4A-4B). Additionally, Transwell Matrigel cell invasion assays revealed that MHCC-97H cells which overexpressed miR-30a-3p markedly reduced cell invasion (Fig. 4C-4D).

### COX-2 is the downstream target of miR-30a-3p in HCC cells

**Figure 5:**
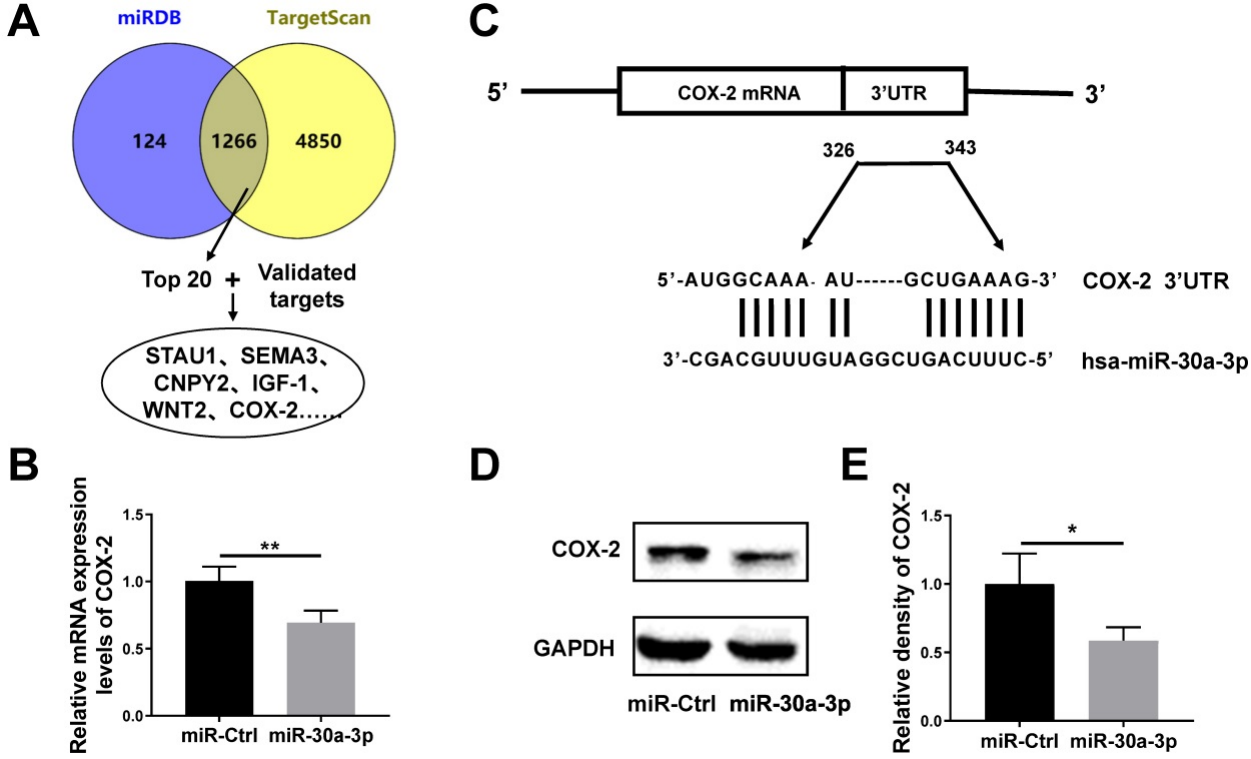
***COX-2* is a direct target gene of miR-30a-3p in MHCC-97H cells.** (A) Strategies for predication and verification of miR-30a-3p's targets. (B) qRT-PCR analysis of COX-2 expression in MHCC-97H cells transfected with miR-30a-3p or miR-Ctrl. (C) miR-30a-3p and its putative binding sequence in the 3'-UTR of COX-2 mRNA. (D-E) Western blot analysis of COX-2 expression in MHCC-97H cells transfected with miR-30a-3p or miR-Ctrl. Data is presented in terms of mean ± SD. *P < 0.05, **P< 0.01.

TargetScan and miRDB databases were used to predict likely miR-30a-3p target genes. Both databases revealed the presence of 1126 shared potential gene targets for miR-30a-3p. We chose the top 20 genes for further investigation based on their total context scores. Meanwhile, we confirmed the existence of 3 genes (COX-2, IGF-1 and WNT2) which are known to be subjected to miR-30a-3p regulation in human cancer cells based on previous studies 9, 19-21 (Fig. 5A). Using qRT-PCR sequencing, we found that amongst those gene candidates, COX-2 was down-regulated in miR-30a-3p overexpressed MHCC-97H cells (Fig. 5B). Using the TargetScan database, we identified two highly-conserved putative binding sites for miR-30a-3p in the 3'-UTRs region of *COX-2* mRNA (Fig. 5C). Dual-luciferase reporter assay was carried out in previous published results, which confirmed COX-2 is one of miR-30a-3p's targets using paired or mutated COX-2 3'UTR oligonucleotides 20. Furthermore, the COX-2 protein level based on western blotting analysis was consistent with qRT-PCR results (Fig. 5D-5E).

### Restoration of COX-2 reversed the anti-tumor effects of miR-30a-3p in MHCC-97H cells

**Figure 6:**
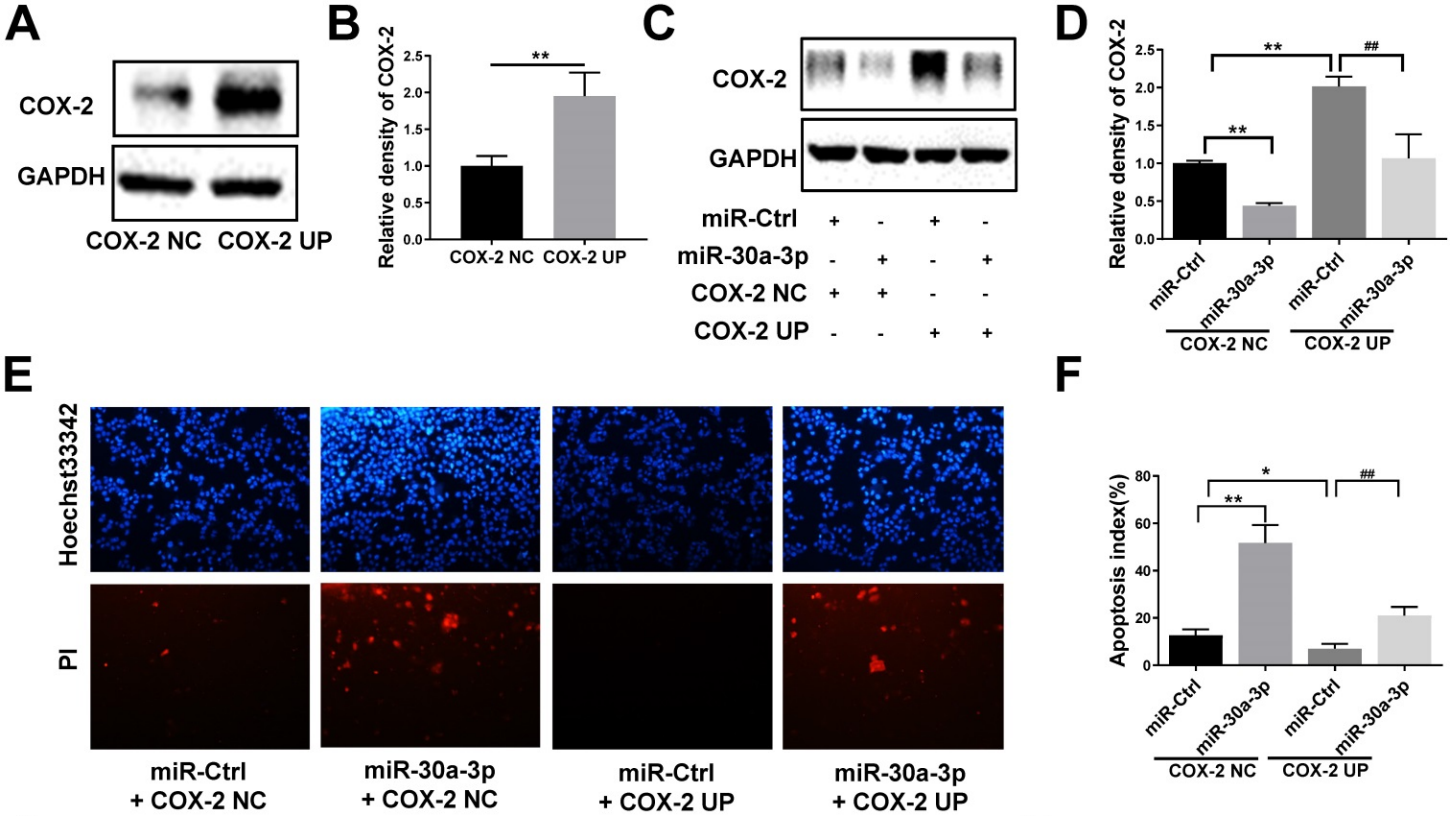

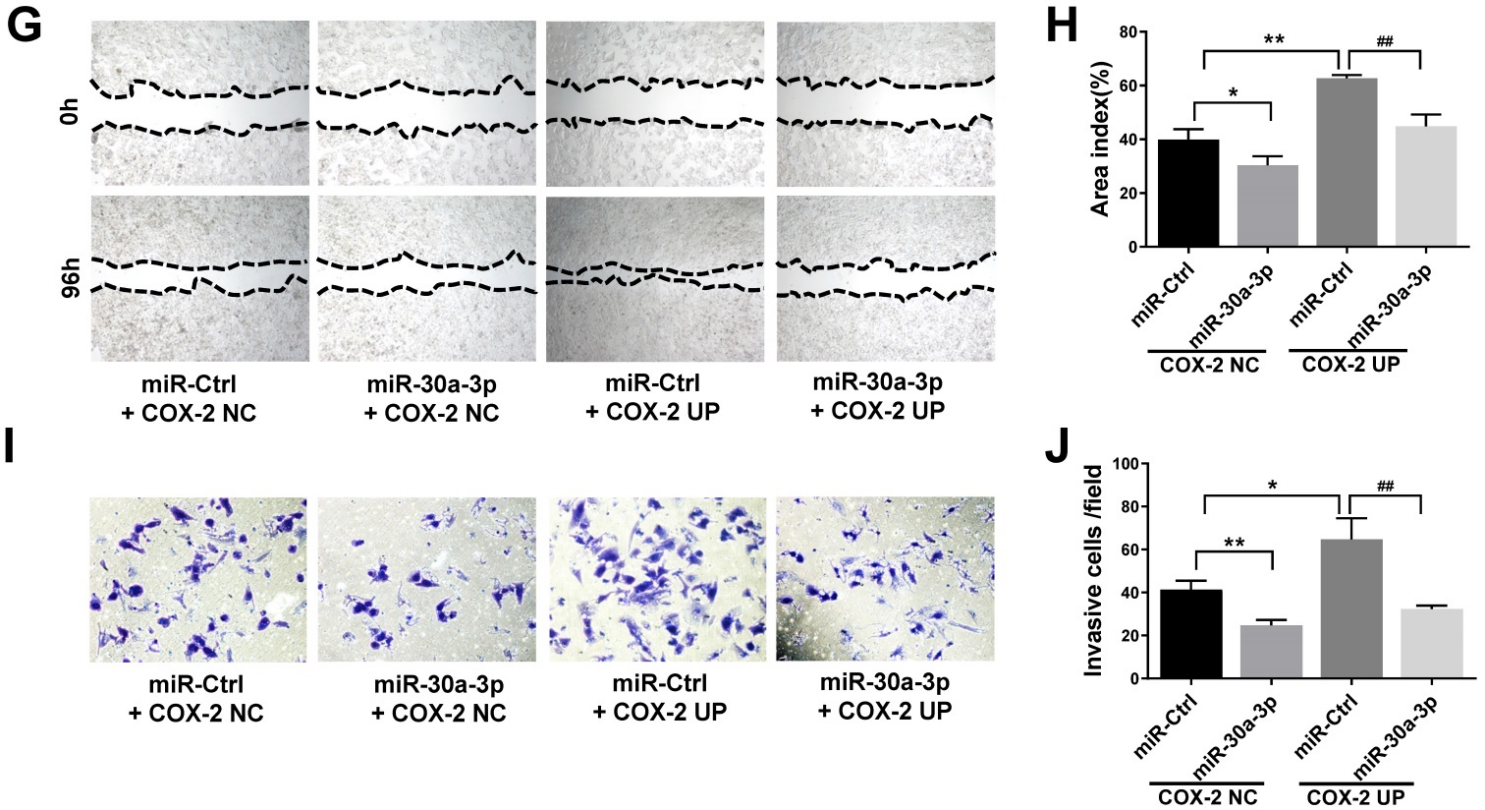
** Restoration of COX-2 attenuated the anti-tumor effects of miR-30a-3p in MHCC-97H cells.** (A-B) COX-2 protein level was significantly increased by transfection of COX-2 expressing lentiviruses. (C-D) MHCC-97H cells which stably transfected COX-2 were transfected with miR-30a-3p expressing lentiviruses or miR-Ctrl. COX-2 protein expression was detected using Western blot. (E-J) Cell apoptosis, invasion, migration assays were subsequently performed. Overexpression of COX-2 attenuated the rates of cell apoptosis (E-F) induced by miR-30a-3p but promoted cell migration (G-H) and invasion (I-J). Data is presented in terms of mean ± SD. *P < 0.05, **P < 0.01 vs. miR-Ctrl+COX-2 NC; ^#^*P* < 0.05, ^##^*P* < 0.01. vs. miR-Ctrl+COX-2 UP.

To further analyze the direct effects of miR-30a-3p on COX-2 during the progression of HCC, MHCC-97H cells were co-transfected with lentiviruses bearing both the *COX-2* and miR-30a-3p genes. Puromycin was used to select stably transfected cells. As predicted, *COX-2* lentiviruses increased COX-2 protein level in MHCC-97H cells. This finding was diminished in cells which were also co-transfected with miR-30a-3p (Fig. 6A-6D). Cell apoptosis induced by miR-30a-3p was also partly reversed in the presence of *COX-2* overexpression (Fig. 6E-6F). Furthermore, the presence of *COX-2* expression significantly enhanced the invasive and migratory abilities of miR-30a-3p overexpressing MHCC-97H cells (Fig. 6G-6J). Together, these findings suggest that miR-30a-3p may act as a tumor suppressor in MHCC-97H cells through targeting COX-2.

### Regulatory effect of miR-30a-3p in the COX-2/PGE2 signaling pathway in MHCC-97H cells

**Figure 7:**
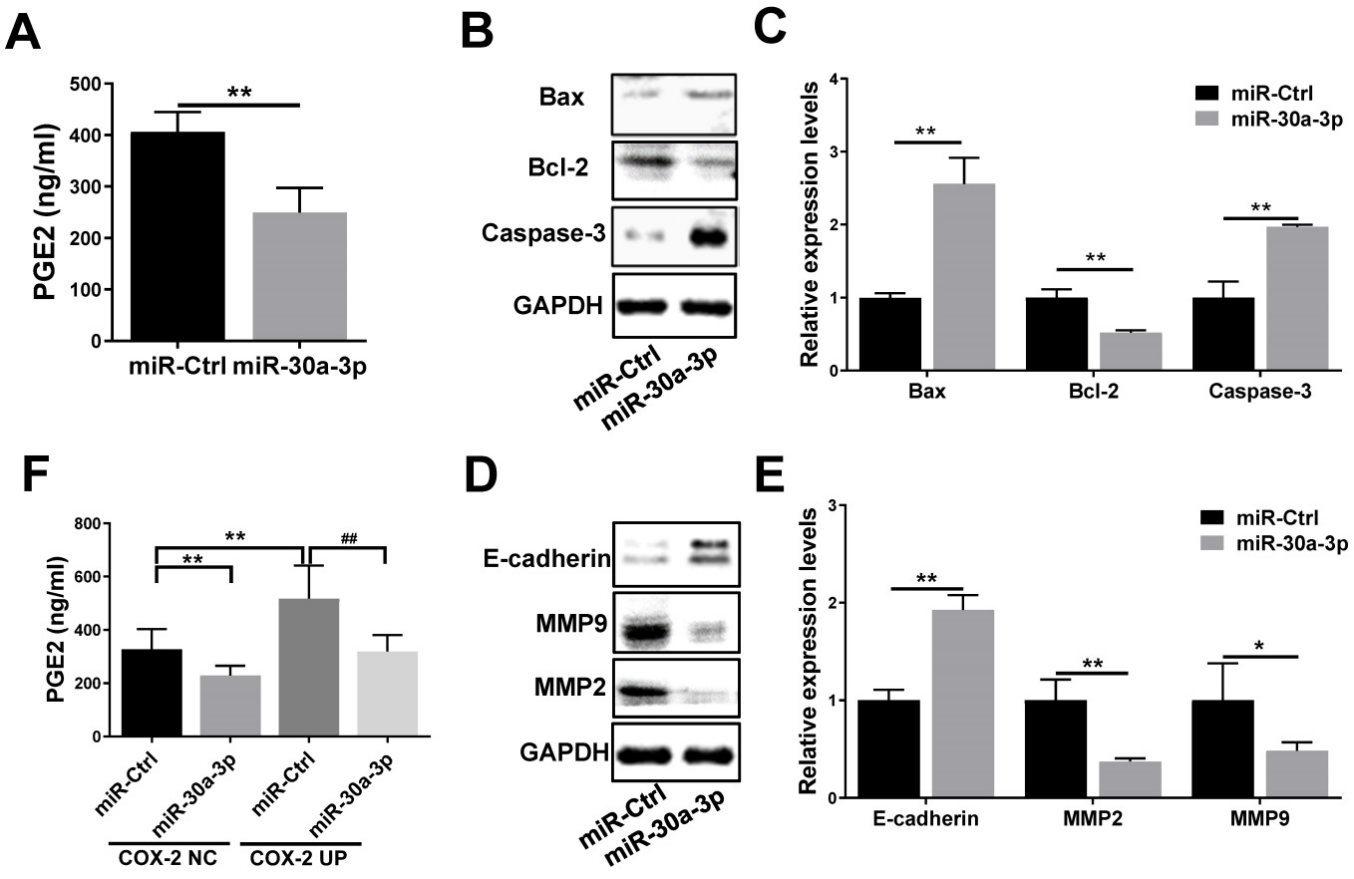

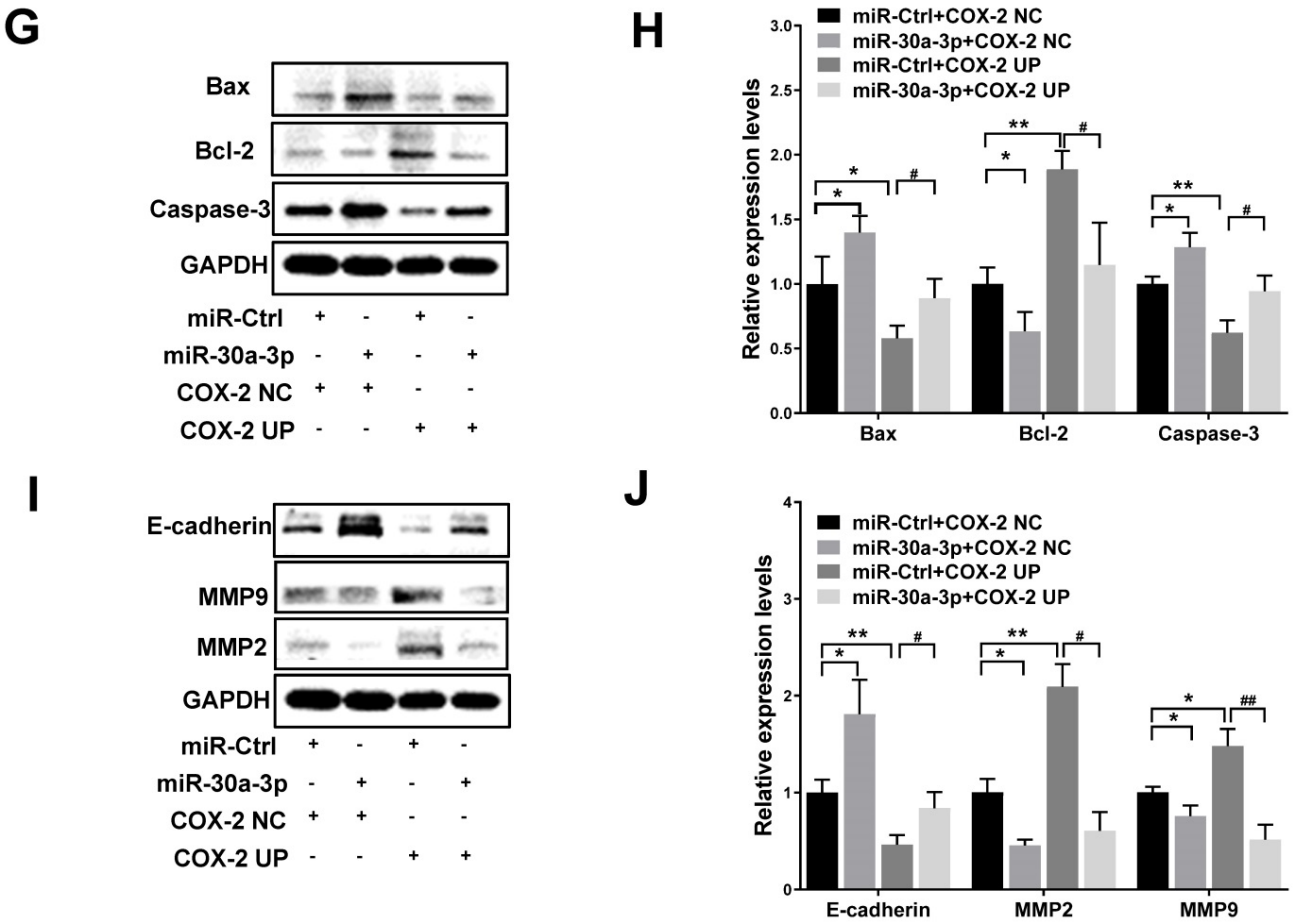
** Inhibitory effect of miR-30a-3p on the COX-2/PGE2 signaling pathway in MHCC-97H cells.** (A) Levels of prostaglandin E2 in cultured supernatants of MHCC-97H cells after transfection with miR-30a-3p or miR-Ctrl were assessed using ELISA assays. (B-E) Effects of miR-30a-3p on PGE2-regulated gene products associated with apoptosis (Bax, Bcl-2 and Caspase-3) and metastasis (E-cadherin, MMP2 and MMP9) were detected by western blotting. (F) Prostaglandin E2 level was measured in miR-30a-3p and COX-2 co-transfection groups. (G-J) Relative protein expressions (Bax, Bcl-2, Caspase-3, E-cadherin, MMP2 and MMP9) were detected upon transfection of miR-30a-3p and COX-2 using Western blotting. Data is depicted in terms of means ± SD. *P < 0.05, **P < 0.01 vs. miR-Ctrl (A-E); *P < 0.05, **P < 0.01 vs. miR-Ctrl+COX-2 NC (F-J); ^#^*P* < 0.05, ^##^*P* < 0.01. vs. miR-Ctrl+COX-2 UP(F-J).

COX-2 is involved in the production of prostaglandin E2 (PGE2), a molecule that has been reported to enhance tumor cell survival and metastasis as well as tumor angiogenesis 22, 23. In order to further understand how miR-30a-3p inhibits HCC progression, we investigated the impact of miR-30a-3p on PGE2 expression. MiR-30a-3p overexpression was capable of suppressing PGE2 level (Fig. 7A). It has been reported that PGE2 inhibited Bcl-2 mediated programmed cell death in colorectal cancer 24. Therefore, we evaluated the expressions of Bcl-2 and its related proteins Bax and Caspase-3 in miR-30a-3p overexpressed MHCC-97H cells. We observed lower level of Bcl-2 and augmented Bax and Caspase-3 level in miR-30a-3p overexpressed MHCC-97H cells (Fig. 7B-7C).

Next, we sought to determine the pathobiological means which reduced the *in vitro* migratory and invasive abilities of MHCC-97H cells with overexpressed miR-30a-3p. It is well known that E-cadherin mediates matrix metalloproteinases (MMPs) expression in multiple highly invasive tumor cells, including MHCC-97H cells 25-27. Here, we found that the expression of E-cadherin is higher in miR-30a-3p overexpressed MHCC-97H cells, which speculatively is a result of suppressed MMP2 and MMP9 expressions (Fig. 7D-7E).

In order to determine whether the effects of miR-30a-3p on HCC were mediated through its activity on the COX-2/PGE2 signaling pathway, stably transfected cells which carried both miR-30a-3p and *COX-2* genes were used to assess the levels of proteins involved in this pathway. As expected, miR-30a-3p appeared to suppress PGE2 (Fig. 7F) and Bcl-2 levels while enhancing Bax and Caspase-3 levels in MHCC-97H cells transfected with COX-2 lentiviruses (Fig. 7G-7H). Similarly, miR-30a-3p overexpression could also markedly increase E-cadherin expression, while inhibiting MMP2 and MMP9 expressions (Fig. 7I-7J).

### Effects of COX-2 inhibition by celecoxib in MHCC-97H cells with overexpressed miR-30a-3p

**Figure 8:**
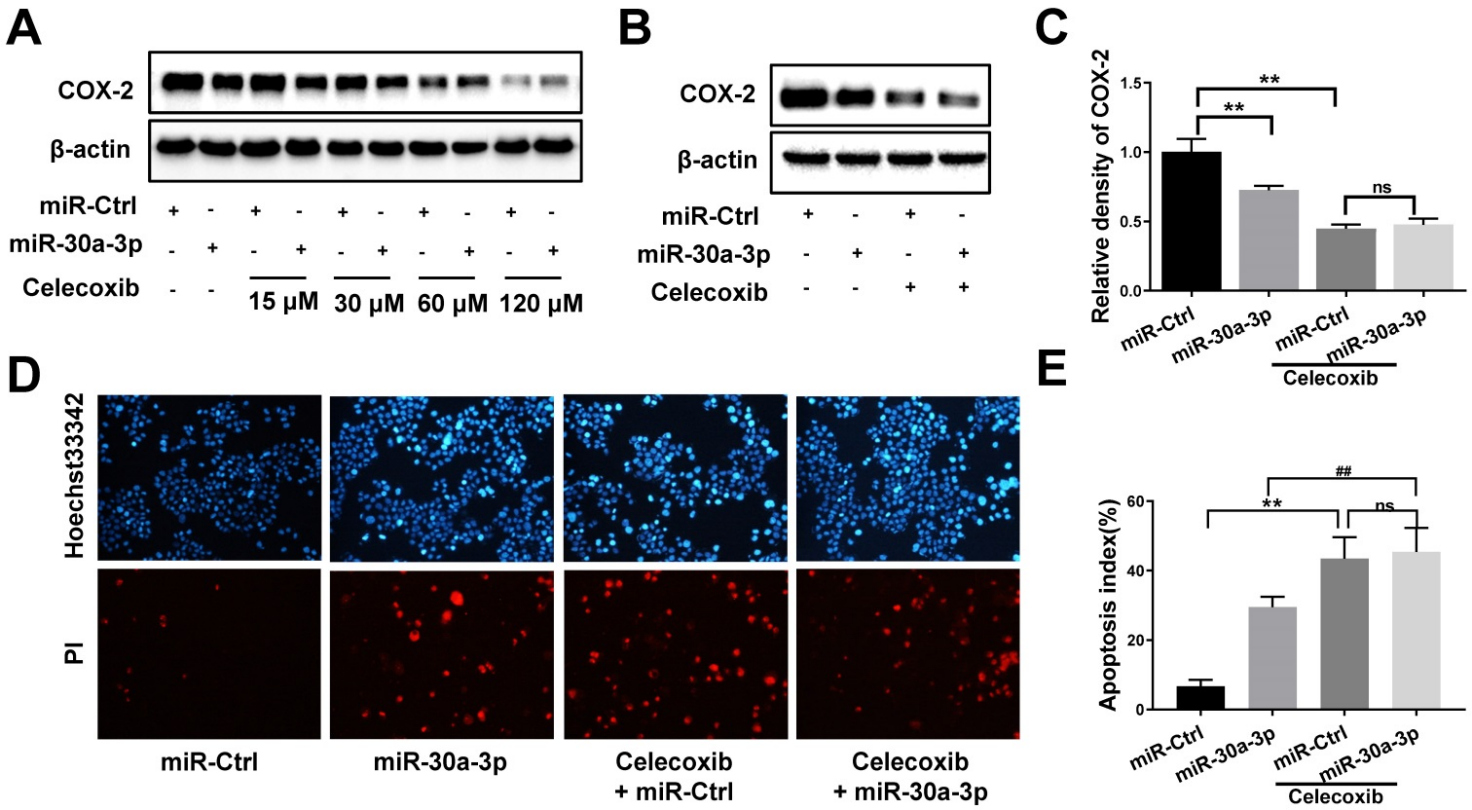

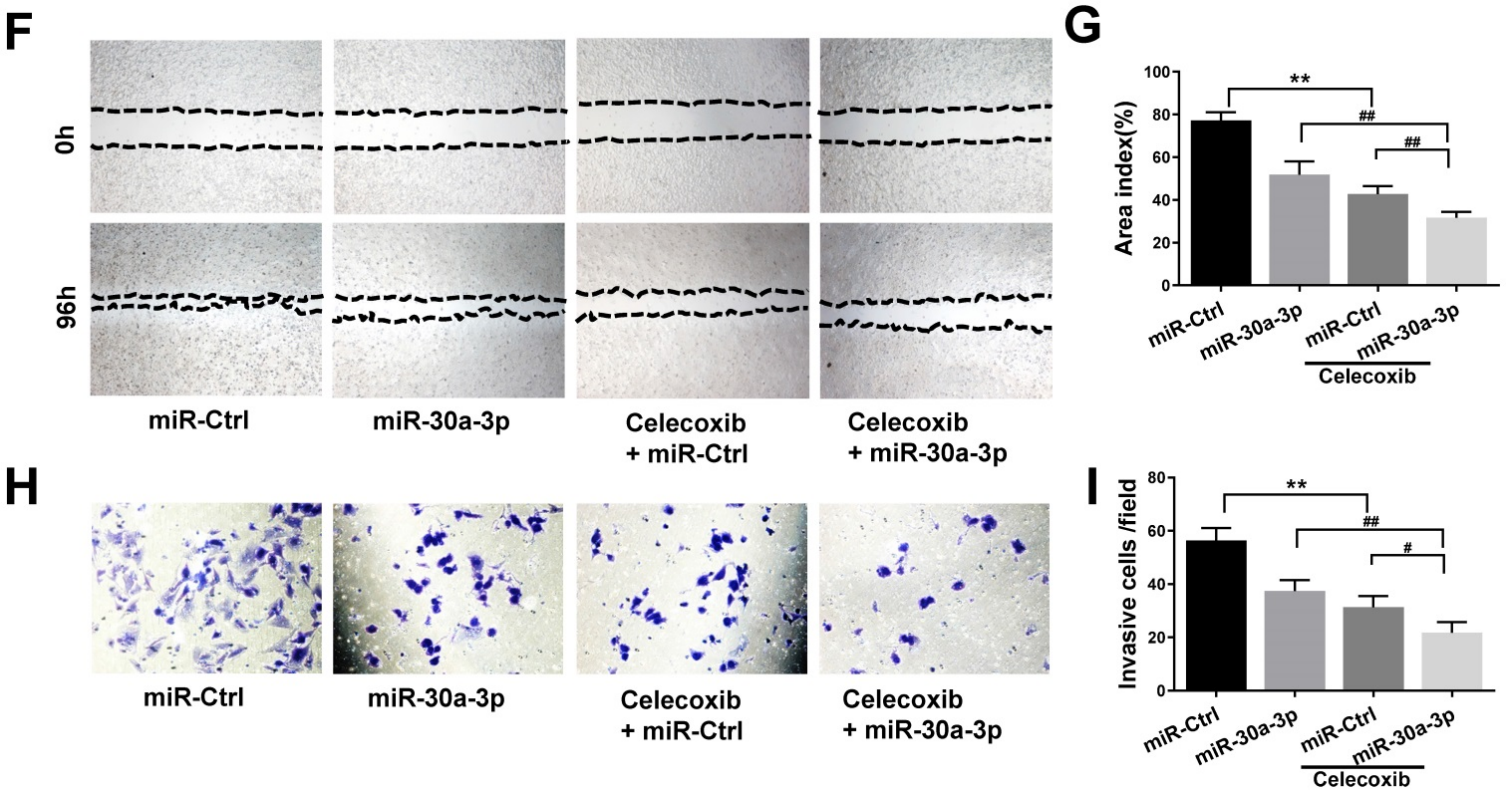
** Effects of COX-2 inhibition by celecoxib in MHCC-97H cells with overexpressed miR-30a-3p.** (A-C) Stably expressing miR-30a-3p MHCC-97H cells were treated with celecoxib. COX-2 protein expression was detected using Western blot. (D-I) Cell apoptosis, invasion, migration assays were subsequently performed. Compared with celecoxib-treated MHCC-97H cells, miR-30a-3p overexpressing cells treated with celecoxib showed similar rates of cell apoptosis (D-E) but a lower rates of cell migration (F-G) and invasion (H-I). Data is depicted in terms of means ± SD. **P < 0.01 vs. celecoxib + miR-Ctrl; ^#^*P* < 0.05, ^##^*P* < 0.01. vs. celecoxib +miR-30a-3p (A-D).

To further determine whether miR-30a-3p acts as a tumor suppressor in MHCC-97H cells through COX-2 targeting, we incorporated the use of a selective COX-2 inhibitor, celecoxib. As expected, celecoxib treatment reduced COX-2 protein levels in both MHCC-97H and miR-30a-3p overexpressing MHCC-97H cells. The miR-Ctrl and miR-30a-3p overexpressing group showed comparable COX-2 level upon exposure to celecoxib at the concentrations of 60μM (Fig. 8A-8C). We proceeded to compare the *in vitro* rates of cell apoptosis, migration and invasion in cells exposed to celecoxib. Interestingly, miR-30a-3p overexpressing MHCC-97H cells did not show increased rates of cell apoptosis compared to control MHCC-97H cells when both were treated with celecoxib (Fig. 8D-8E). Instead, celecoxib-treated groups exhibited reduced cell migration and invasion in contrast to untreated MHCC-97H cells (Fig. 8F-8I). However, in comparison to control MHCC-97H cells, miR-30a-3p overexpressing MHCC-97H cells treated with celecoxib demonstrated a higher degree of inhibitory effects, suggesting that miR-30a-3p may work on several targets in MHCC-97H cells.

## Discussion

Downregulation of miR-30a-3p has been frequently reported in several types of tumors, including renal, lung, gastric and liver cancers 9, 10, 17, 28, 29. These studies support the essential role of miR-30a-3p in tumor growth, proliferation, apoptosis, invasion and migration. The current investigation analyzes the function and molecular mechanisms of miR-30a-3p in the MHCC-97H cell line. Consistent with previous published results, we found that miR-30a-3p was downregulated in MHCC-97H cells. Meanwhile, augmented miR-30a-3p expression appeared to stunt *in vitro* and *in vivo* MHCC-97H cell growth, a phenomenon which we attribute to increased rates of cell apoptosis. MiR-30a-3p was also observed to hinder HCC cell invasion and migration, which have previously been reported in studies of this molecule in the HCC Bel-7402 cell line 17. Furthermore, we demonstrated that miR-30a-3p appeared to directly target *COX-2*, as demonstrated by reduced cellular *COX-2* mRNA and protein levels in cells with overexpressed miR-30a-3p. Interestingly, an overexpression of *COX-2* attenuated the impact of miR-30a-3p on cell migration and growth. In summary, these results strongly suggest that miRNA-30a-3p exerts a tumor suppressing effect on the MHCC-97H cell line through *COX-2* targeting.

COX-2 expression and the abundance of its enzymatic product PGE2 are crucial in aiding the development of malignant cells 22. Several investigations have documented that an overexpression of *COX-2* or exogenous PGE2 addition contributed to enhanced angiogenesis 30, apoptosis resistance 31 and increased invasion and metastasis across several tumors 32, 33. Our results revealed that miR-30a-3p suppressed COX-2 and PGE2 expressions in MHCC-97H cells, which has also been reported in gastric cancer 20. Bcl-2 is associated with apoptosis of cancer cells and is known to be regulated by the COX-2/PGE2 signal pathway 34, 35. During cell apoptosis, Bcl-2 competes with the expression of apoptotic gene Bax, and inhibits cell apoptosis by modulating mitochondrial cytochrome c release which in turn inhibits Caspase-3 activation 36, 37. Our findings revealed that miR-30a-3p induced cell apoptosis, as evidenced by downregulation of Bcl-2 as well as upregulation of Bax and Caspase-3 expressions. Nevertheless, restoring COX-2 reversed the changes noted in Bcl-2, Bax and Caspase-3 expressions. Based on these findings, we propose that miR-30a-3p regulates MHCC-97H cell death through COX-2/PGE2 and Bcl-2/Bax/Caspase3 signal pathway.

Distant metastasis is a common occurrence in patients with HCC and represents a significant modifier of overall patient survival. Previous studies revealed that MMP-2/9 promoted tumor metastasis and invasion by degrading extracellular matrix proteins 38, 39. In addition, E-cadherin has been reported as an inhibitory factor of invasion and metastasis in several highly invasive cancers and is thought to impact this effect by modifying MMPs expression 21, 25. Our results demonstrate that MMP-2 and MMP-9 expressions are suppressed by upregulated miR-30a-3p level, while *COX-2* co-transfection reverses this effect. E-cadherin expression level is also noted to be raised following increased miR-30a-3p level whereas it was suppressed after COX-2 overexpression. We therefore hypothesize that miR-30a-3p exerts an inhibitory role on MHCC-97H cell migration and invasion by modulating the COX2/PGE2-E-cadherin-MMP2/9 pathway.

To further examine the relationship between COX-2 and miR-30a-3p, celecoxib was used as a selective COX-2 inhibitor. Compared with celecoxib-treated MHCC-97H cells, miR-30a-3p overexpressing cells treated with celecoxib demonstrated similar rates of cellular apoptosis but a less degree of metastasis *in vitro*, which suggest that COX-2 is indispensable in miR-30a-3p induced cell apoptosis. However, inhibiting COX-2 appeared to slow metastasis in miR-30a-3p overexpressing cells. It is well known that miRNAs often target the expressions of multiple genes, and a single gene may be simultaneously regulated by several different miRNAs. Therefore, we conclude that miR-30a-3p may target other metastasis-related gene targets in MHCC-97H cells which have yet been discovered.

Our experiments uncover a significant role of miR-30a-3p in modulating the malignant progression of MHCC-97H HCC cells. This effect is likely the result of *COX-2* targeting. Taken together, miR-30a-3p holds potential as a novel therapeutic target in HCC management.

## Supplementary Material

Supplementary table S1.
