# MicroRNA-30a-3p acts as a tumor suppressor in MHCC-97H hepatocellular carcinoma cells by targeting COX-2: Erratum

**DOI:** 10.7150/jca.78550

**Published:** 2022-11-03

**Authors:** XueMei Yang, JiaLing Sun, HaiTao Sun, Bin Wen, MingJia Zhang, HaiYan An, WeiCong Chen, WenTing Zhao, XiaoDan Zhong, ChunYu He, Jie Pang, SongQi He

**Affiliations:** 1 School of Traditional Chinese Medicine, Southern Medical University, Guangzhou, Guangdong, China.; 2 Department of Traditional Chinese Medicine, The Air Force Hospital Of Southern Theater Command, Guangzhou, Guangdong, China.

We recently noticed a mistake in Figure 6E, where “miR-Ctrl+Cox-2 NC” group overlapped with “miR-30a-3p+Cox2 up” group. The correct figure is provided in the following. This error does not affect the results and conclusions of the article.

The Western Blot bands of GAPDH in Figure 7G and 7I were reused due to the WB samples being the same. To distinguish cell death-related proteins and cell metastasis-related proteins, we put the results in 7G and 7I respectively. The proteins were separated in two gels with the same operation at the same time. Due to some protein's molecular weights being very close (Bax-21kD, Bcl2-26kD, Caspase3-32kD, GAPDH-37kD, E-cadherin-135kD, MMP2-72kD, MMP9-92kD), we only got one band for GAPDH incubation. GAPDH was used twice as an internal control in this figure. To prevent misunderstanding we replace the picture in Figure 7I.

All the authors of the paper have agreed to this correction. The authors apologize for any inconvenience that the errors may have caused.

## Figures and Tables

**Figure 6:**
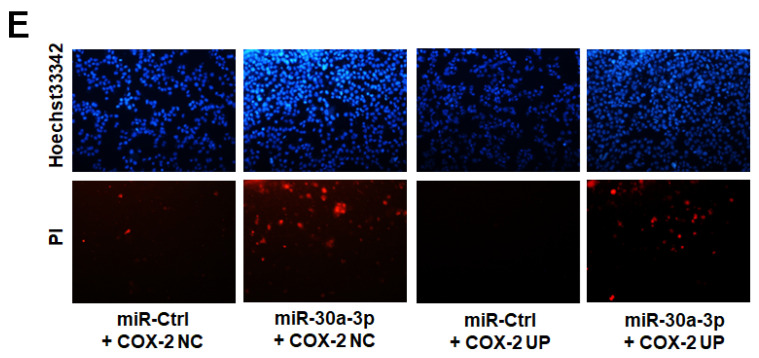
** Restoration of COX-2 attenuated the anti-tumor effects of miR-30a-3p in MHCC-97H cells.** (E) Cell apoptosis were subsequently performed. Overexpression of COX-2 attenuated the rates of cell apoptosis induced by miR-30a-3p. Data are means ± SD. ^*^P < 0.05, ^**^P < 0.01 vs. miR-Ctrl+COX-2 NC; ^#^*P* < 0.05, ^##^*P* < 0.01. vs. miR-Ctrl+COX-2 UP.

**Figure 7:**
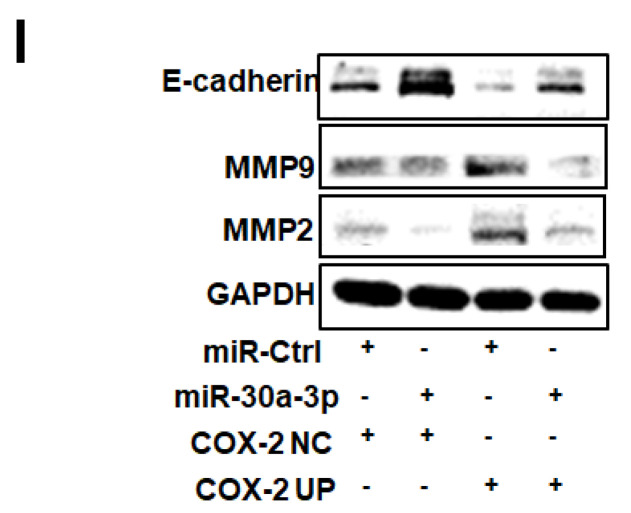
** Inhibitory effect of miR-30a-3p on the COX-2/PGE2 signaling pathway in MHCC-97H cells.** (I) Relative protein expressions (E-cadherin, MMP2 and MMP9) were detected upon transfection of miR-30a-3p and COX-2 using Western blotting. Data is depicted in terms of means ± SD. ^*^P < 0.05, ^**^P < 0.01 vs. miR-Ctrl+COX-2 NC; ^#^*P* < 0.05, ^##^*P* < 0.01. vs. miR-Ctrl+COX-2 UP.

