# Supplementary material for: MicroRNA-30a-3p acts as a tumor suppressor in MHCC-97H hepatocellular carcinoma cells by targeting COX-2
Source: J Cancer. 2021 May 10;12(13):3945–57. doi: 10.7150/jca.52298 (PMC8176251; doi:10.7150/jca.52298)
Supplement: Supplementary file 1 — Supplementary table S1. [file jcav12p3945s1.pdf]

1. The primers of predicted target gene by miR-30a-3p

Primer name	Sequence (5'to3')
**hsa-STAU1-F**	GCCTGTTGACCCTTACTCTCG
**hsa-STAU1-R**	TGGAAATGGGTAAAAGTACCT
**hsa-SEMA3E-F**	AGGCAGGGACCTTGTATATTCC
**hsa-SEMA3E-R**	TGTACTCGGCCAGTGTATCTC
**hsa-CNPY2-F**	TCTGGTGGATGAACTAGAATGGG
**hsa-CNPY2-R**	TGTTTGGGGGAACAGTAACAGT
**hsa-CDC73-F**	CTGGCCCAAGAATGTGAAGAC
**hsa-CDC73-R**	GCACGTCGGACATAAACAGGAT
**hsa-UBE2G1-F**	AGGTGGTGTTTTTAAGGCTCATC
**hsa-UBE2G1-R**	CATTTGGGTGCCAGATTTCTGTA
**hsa-PANK3-F**	TTTTGGCCGAAGAGGGAACTT
**hsa-PANK3-R**	TAGCACCGTCTGCAATGTTGA
**hsa-PDK1-F**	CTGTGATACGGATCAGAAACCG
**hsa-PDK1-R**	TCCACCAAACAATAAAGAGTGCT
**hsa-SEMA3C-F**	TTTGCGTGTTGGTTGGAGTAT
**hsa-SEMA3C-R**	TCCTGTAGTCTAAAGGATGGTGG
**hsa-PCDH17-F**	GCACGGTGATCGGGAACAT
**hsa-PCDH17-R**	GCGCTGCTTGGTGTAGAGG
**hsa-RIF1-F**	AGGGCAGACTGACGCTTAC
**hsa-RIF1-R**	GCAGCACTACTCAGCTCCG
**hsa-TOB1-F**	ATGCCCATAAGTGACCCAGC
**hsa-TOB1-R**	GTAGAGCCGAACTTGGTGGC
**hsa-ROR1-F**	TGCCAGCCCAGTGAGTAATCT
**hsa-ROR1-R**	GCCAATGAAACCAGCAATCTG
**hsa-WNT2-F**	GGGGCACGAGTGATCTGTG
**hsa-WNT2-R**	GCATGATGTCTGGGTAACGCT
**hsa-FZD2-F**	GTGCCATCCTATCTCAGCTACA
**hsa-FZD2-R**	CTGCATGTCTACCAAGTACGTG
**hsa-IGF-1 a-F**	ACTGAGCTCTGATGAGTTAATGTGCAACC
**hsa-IGF-1 a-R**	ACTCTCGAGCCTCTGATCCTTGAGGTGA
**hsa-IGF-1 b-F**	ACTGAGCTCGACCATACTGGATACTTAGGTC
**hsa-IGF-1 b-R**	ACTCTCGAGCTAAGCCTTCTCCCAAGTG
**hsa-IGF-1 c-F**	ACTGAGCTCGAAACCTCTCACAGATAAGAC
**hsa-IGF-1 c-R**	ACTCTCGAGCCTACTTTACATCAGTGCA
**hsa-PTEN-F**	TGGATTCGACTTAGACTTGACCT
**hsa-PTEN-R**	GGTGGGTTATGGTCTTCAAAAGG

**hsa-AKT3-F**	TGTGGATTTACCTTATCCCCTCA
**hsa-AKT3-R**	GTTTGGCTTTGGTCGTTCTGT
**hsa-COX2-F**	GCACCCCGACATAGAGAGC
**hsa-COX2-R**	CTGCGGAGTGCAGTGTTCT
**hsa-ERBB4-F**	GTCCAGCCCAGCGATTCTC
**hsa-ERBB4-R**	AGAGCCACTAACACGTAGCCT
**hsa-p53-F**	AACTGCGGGACGAGACAGA
**hsa-p53-R**	AGCTTCAAGAGCGACAAGTTTT
**hsa-MeCP2-F**	TGACCGGGGACCCATGTAT
**has-MeCP2-R**	CTCCACTTTAGAGCGAAAGGC
